# Use of muscular flaps for the treatment of hip prosthetic joint infection: a systematic review

**DOI:** 10.1186/s12891-021-04945-8

**Published:** 2021-12-23

**Authors:** Giuseppe Rovere, Domenico De Mauro, Marco D’Orio, Camillo Fulchignoni, Maria Rosaria Matrangolo, Carlo Perisano, Antonio Ziranu, Elisabetta Pataia

**Affiliations:** Department of Orthopedics and Traumatology, Fondazione Policlinico Universitario A. Gemelli IRCSS, Università Cattolica Del Sacro Cuore, Largo Agostino Gemelli 8, 06168 Roma, Italy

**Keywords:** Arthroplasty, Hip replacement, Joint infection, Hip prosthesis, Muscular flaps

## Abstract

**Background:**

Deep periprosthetic infection after total hip arthroplasty (THA) is a serious and challenging complication for the orthopedic surgeon. Muscular flaps may represent a valid management option for the treatment of this condition. We present a systematic literature review about the use of muscular flaps for the treatment of hip prosthetic joint infection.

**Methods:**

The review is reported according to the Preferred Reporting Items for Systematic Reviews and Meta-Analyses (PRISMA) guidelines. Seventy-seven articles, out of 279 titles, were considered eligible for the full-text analysis. Finally 15 studies that met inclusion criteria were included in this review.

**Results:**

Overall, 210 patients (49% males, 48.6% females and 2.4% not reported) suffering from THA infection treated with muscular flaps were collected. The mean age was 69.6 years. Mean follow-up, reported in all studies, was 3.3 years.

The results presented by the different authors, highlight the effectiveness of muscular flaps for the treatment of periprosthetic infection, in terms of function, limb salvage, prevention of the recurrences, cost-effectiveness, and quality of life postoperatively.

**Conclusions:**

Muscle flaps provide an excellent management option for patients with persistent infection after total hip arthroplasty.

## Background

Total hip arthroplasty (THA), is one of the most reliable and successful surgical procedures in orthopedic surgery with high clinical outcomes in patients with symptomatic ostheoarthritis [1]. Deep periprosthetic joint infection, although uncommon, is the most serious and challenging complication for the orthopedic surgeon. It occurs in approximately 0.57 to 2.23% of hip replacements [2] causing significant physical and psychological morbidity in affected patients.

The correct surgical approach for the treatment of infected hip arthroplasty remains a matter of controversy in the literature. Conventional treatment with debridement and antibiotics is usually the first step. One or two-stage revision hip arthroplasty is considered the treatment of choice with a high rate of success in controlling infection in non-responding patients (80-100%) [3]. The Girldestone resection arthroplasty technique is the most used option in those patients with deep recurrent infections [4, 5] but even with this approach, 20% of infections may persist [6, 7]. In recalcitrant cases, after Girldestone resection, open fibrotic wounds with large unhealed dead spaces are left in the acetabular cavity as well as in the area of the old femoral neck and greater trochanter [8]. These dead spaces are an excellent breeding ground for the perpetuation of the infection [8].

Integumentary defects after hip replacement are difficult to manage, especially if the infection is deeper than expected and the prosthesis is already involved or if the bone or prosthesis are exposed [9, 10].

The orthopedic and plastic surgeons should work as a joint team for the correct management of prosthetic infection, especially in severe cases whose evolution would otherwise be, in many cases, amputation [11, 12].

Flaps have been widely used in orthopedic surgery for the management of congenital, tumoral and infectious diseases and on the basis of anatomical content they can be divided into: skin flaps, muscle and myo-cutaneous flap and fascia or fascio-cutaneous flap [13].

Vascularized muscle flaps may fill the unhealed dead spaces with healthy and well vascularized tissue. This allows covering of the wound and provides adequate local blood supply with faster and complete absorption of antibiotics.

Discordant data on the use of muscle flaps for hip replacement infection are reported in the literature. For this reason, we report a systematic review of the literature on the role of muscular flaps for the treatment of hip prosthetic joint infection, on the real impact of this procedure in THR infections and most of all, on the comparison of the experiences currently available in the literature.

## Methods

### Study setting and design

**Fig. 1:**
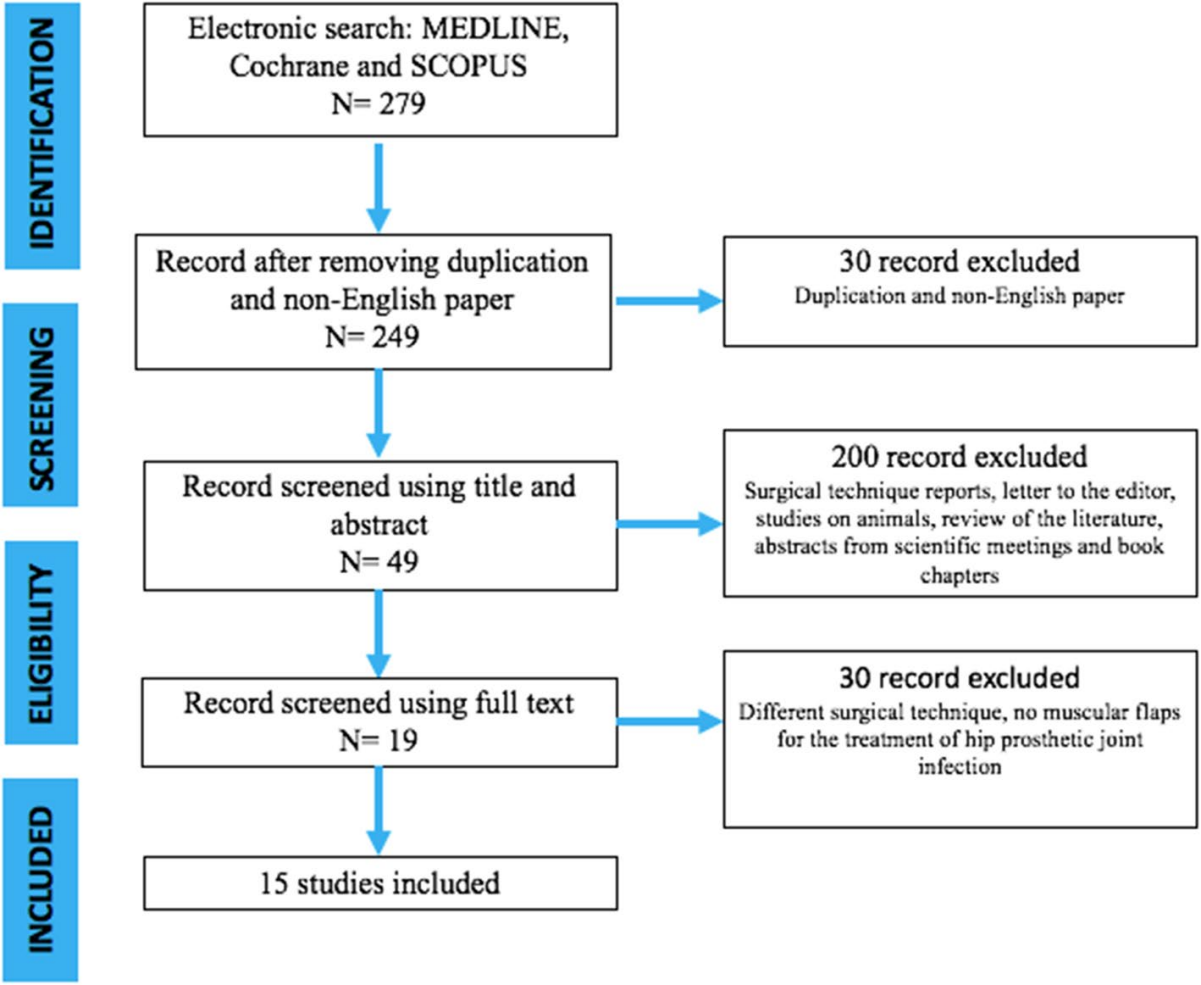
PRISMA Flow-chart

The present investigation represents a systematic literature review reported according to the Preferred Reporting Items for Systematic Reviews and Meta-Analyses (PRISMA) guidelines (Fig. 1).

### Review questions

The review questions were formulated following the PICO scheme (population (P), intervention (I), comparison (C), and outcome (O) as follows:

- Do patients suffering from periprosthetic hip infection (P) report better clinical outcomes (C), in term of complete healing rate (O), when treated through muscular flaps (I)?
- Are muscular flaps useful (I) for the management of prosthetic infection (O)?

### Inclusion and exclusion criteria

In this review we considered the studies published as full-text articles in indexed journals, which investigated the value of muscular flaps for the management of hip prosthetic infection. Only articles written in English with available abstract were included. No publication date limits were set. Surgical technique reports, expert opinions, letter to the editor, studies on animals, unpublished reports, cadaver or in vitro investigations, review of the literature, abstracts from scientific meetings and book chapters were excluded from the present review.

### Search strategy and study selection

Scopus, Cochrane Library, MEDLINE via PubMed and Embase were searched using the keywords: “muscular flaps”, “Vastus lateralis muscle flap”, “rectus femoris muscle flaps”, “muscular”, “flaps”, “hip prosthetic joint infection”, “Infected Total Hip Arthroplasty”, “infected hips”, “two-stage revisions”. and their MeSH terms in any possible combination. The reference lists of relevant studies were screened to identify other studies of interest. The search was reiterated until March 15, 2021.

### Data extraction and analysis

Two independent reviewers (M.D and D.D.M) collected the data from the included studies. Any discordances were solved by consensus with a third author (G.R.). For each study included in the present analysis, the following data were extracted: Year, Types of Research Studies, demographic features, sex, age, diagnosis, previous hip surgery, pathogens, treatment performed, possible complications and outcomes, and follow-up. Numbers software (Apple Inc., Cupertino, CA) was used to tabulate the obtained data. Categorical variables are presented as frequency and percentages. Continuous variables are presented as means and standard deviation. Only one decimal digit was reported and was rounded up.

## Results

After screening 279 articles by title and abstract, 77 were considered eligible for the full-text analysis. Sixty studies were excluded because they did not fulfill inclusion criteria. Finally 15 studies (Table 1) that met inclusion criteria were included in this review (Fig. 1). All these studies had a retrospective descriptive design, 4 were case reports [14–17] and 11 were case series [3–8, 18–25].

**Table 1:** Selected articles

Article	Type of study	Number of patients	Type of flap	Mean follow-up (years)
Arnold, 1983 [19]	Retrospective	7	6 rectus femoris, 3 vastus lateralis	2.5
Jones, 1991 [18]	Case Series	5	3 rectus abdominis, 1 latissimus dorsi free flap, 1 vastus lateralis	2.1
Meland, 1991 [7]	Retrospective	27	23 rectus femoris, 8 vastus lateralis, 1 tensor fasciae latissimus, 2 combined latissimus dorsi-serratus anterior free-tissue transfer	6.4
Lewis, 1994 [14]	Case Report	1	1 tensor fasciae latissimus	4
Windle, 1996 [21]	Case Series	3	3 rectus abdominis	5.3
Lee, 1996 [20]	Retrospective	7	7 vastus lateralis	2.5
Ross, 1998 [15]	Case Report	1	1 rectus abdominis	1
Ikeda, 2001 [22]	Case Series	1	1 vastus lateralis	2.8
Gusenoff, 2002 [16]	Case Report	4	4 vastus lateralis	n.a.
Huang, 2005 [23]	Case Series	4	4 vastus lateralis	1.6
Shieh, 2007 [8]	Case Series	1	1 vastus lateralis	2
D’Ettorre, 2010 [17]	Case Report	2	2 vastus laterali	4
Suda, 2010 [3]	Retrospective	119	119 vastus lateralis	2.6
Choa, 2011 [24]	Retrospective	24	20 rectus femoris, 5 vastus lateralis	3.9
Ricciardi, 2017 [26]	Retrospective	4	4 gluteus maximus	n.a.

Overall, 210 patients (49% males, 48.6% females and 2.4% not reported) suffering from periprosthetic hip infection treated with muscular flaps were collected. Patients had a mean age at diagnosis ranging from 31.0 to 72 years, and the mean follow-up range was 86 days - 9.0 years (Table 2).

**Table 2:** Epidemiological data

**Number of patients**	**210**
- **Male**	103 (49.0%)
- **Female**	102 (48.6%)
- **Not reported**	5 (2.4%)
**Mean Age (years)**	69.6 (SD 10.8)
**Mean Follow-up (years)**	3.3 (SD 1.5)
**Mean number of surgeries before flap**	4.7 (SD 2.4)
**Number of flaps**	220
- **Vastus lateralis**	155 (70.4%)
- **Rectus femoris**	49 (22.3%)
- **Rectus abdominis**	7 (3.2%)
- **Gluteus maximus**	4 (1.8%)
- **Latissimus dorsi**	3 (1.4%)
- **Tensor fascia latissimus**	2 (0.9%)

Pathogens responsible for the infections were reported in 10 studies (66.6%). The most common organism was *Staphylococcus aureus*, which was detected in 9 studies [3–8, 16, 17, 22–25] followed by Enterococcus (in 6 studies) [3, 7, 16, 22, 23] and *Pseudomonas Aeruginosa* (in 5 studies) [3, 7, 16, 20, 22]. Only two papers specified antibiotic therapy administered [17, 24]. Choa et al. [24] described that Vancomycin 1 g bis in die (bid), and Meropenem 500 mg to 1 g, three times a day (tid) were intra-operative administered, after deep microbiological sampling. D’Ettorre et al. [17] otherwise reported, in one of their two cases, the use of vancomycin 1 g and rifampicin 600 mg every 12 h after surgery, followed by teicoplanin therapy, three times per week at dosages of 800 + 800 + 1200 mg/dl, combined with rifampicin and minocycline, on discharge. In the second case they administered the same therapy on discharge preceded by intravenous teicoplanin on the day of surgery at a dose of 6 mg/Kg/day plus rifampicin and minocycline.

As shown in Table 1 different types of muscular flap were reported for treatment of hip prosthetic joint infection: vastus lateralis muscle flap was used in 155 patients; rectus abdomen in 7 patients; rectus femoris in 49 patients; tensor fascia lata in 2 patients; latissimus dorsi in 3 patients and gluteus maximus in 4 patients. In all of the reported series, muscular flaps successfully healed the deep infection in the treated patients (Table 3).

**Table 3:** Results and complications

*Type of flap*	*Rate of complete healing at last follow-up*	*Complications*
*Vastus lateralis*	99.3%	20 hematoma (12.9%), 3 erysipela (1.9%), 3 recurrences (1.9%), 2 flap failure needing revision (1.3%), 1 flap needed partial debridment (0.6%)
*Rectus femoris*	97.9%	1 flap failure needing revision (2.0%), 1 sterile seroma drained non surgically (2.0%)
*Rectus abdominis*	100%	1 incisional hernia (14.3%)
*Gluteus maximus*	100%	2 recurrent drainages (50%)
*Latissimus dorsi*	100%	None
*Tensor fascia latissimus*	100%	None

Most authors report complete wound healing and infection resolution at last follow-up, without specifying the functional outcome in their patients affected by recalcitrant THA infections [7, 14, 16–23]. On the other hand Arnold et al. [19] describe how all their 7 patients were healed and able to bear weight at final follow-up. Also Ross et al. [15] stresses that their single patient, fully healed, at 3 months post operative was ambulating with the aid of a cane and at 1 year was walking unaided.

Lee et al. [20] in 3 out of 7 cases had a recurrent infection after the flap procedure, healed with antibiotics. Windle et al. [21] reported in 1out of 3 cases an incisional hernia on the donor site; Arnold et al. [14] described 1 case of partial debridement of the transposed muscle and a sterile seroma, developed 2 weeks after hospital discharge, and treated by needle aspiration.

## Discussion

This review analyzed the current data regarding the use of muscular flaps for the treatment of hip prosthetic joint infection. Delayed wound healing, wound dehiscence and infection, associated with the loss of soft tissue are potentially catastrophic complications of hip replacements [4]. Fibrosis of the soft tissues and persistence of large, deep cavities make secondary closure usually doomed to failure [24]. Breakdown of soft tissue over a prosthetic joint replacement leaves the prosthesis and surrounding bone susceptible to exposure, infection, and potential loss of both joint and limb. For large wounds, healing may be difficult or impossible, requiring pedicled or free muscle flaps to achieve adequate coverage [7–29].

There is growing evidence supporting possible benefit of muscle flaps coverage for the treatment of persistent infection, compared with (or after failing of) conventional techniques such as one or two stage revision, or debridement and antibiotic therapy [26, 30]. Muscle flaps have the main advantage of having independent intrinsic blood supply and of being malleable conforming to wounds with irregular contours. Mathes and Nahai [31] classified muscular flaps, according to the pattern of vascular supply in five types: type I muscles, have a single dominant vascular pedicle (eg. tensor fascia lata); type II muscles, like the gracilis, have a dominant pedicle and minor/segmental pedicles; type III muscles, have two dominant pedicles, only one of which is necessary to supply the muscle (for example, rectus abdominis and gluteus maximus); type IV muscles, like sartorius or tibialis anterior, have a segmental blood supply with no dominant pedicle and type V muscles, like pectoralis major or latissimus dorsi muscle, have a dominant pedicle and secondary segmental pedicles. The latter can be supplied by secondary pedicles if the dominant pedicle gets sacrificed. The different anatomical characteristics of muscular flaps, make their use versatile [32–35]. They may be used locally (such as gluteus medius, tensor fascia lata, rectus femoris and rectus abdominis) remaining attached to their blood supply, or for distant reconstructions as free tissue transfer (like latissimus dorsi), requiring microvascular anastomosis.

Ricciardi et al. showed that gluteus maximus has the main advantage of non-causing functional impairment because its origin, insertion and innervation are preserved [29]. On the other hand, vastus lateralis flap is easy to harvest, as the size of this muscle and its constant blood supply make it ideal for filling the infected cavity after resection arthroplasty. The muscle has two main anterior proximal nutrient vessels: normally these vessels are not damaged by previous surgery to the hip. Used as an island flap, this muscle has adequate range and sufficient volume to fill the infected cavity completely [3]. The rectus abdominis flap has the main advantage of preserving the lower extremity strength unlike most of the local flaps although the muscle could be hypotrophic or fibrotic expecially in elderly patients [15, 21]. Free latissimus dorsi miocutaneous flap is the best choice in case of a very large dead space, even though it is sometimes only just sufficient to fill these large cavities. Despite the increasing of surgery time and difficulties due to microsurgical procedure, this remains the best option in such extended loss of substance and after previous local flaps failure [18].

For each muscle some limitations have been described. The main limitation of rectus femoris and gluteus medius – tensor fascia lata flaps, is the loss of important stabilizers of the hip and knee joint. The main disadvantages of rectus abdominis flaps are at first the need of a second skin incision at the donor site and also that it requires the disruption of the acetabulum for transpelvic transposition. Moreover the rectus abdominis may be too thin and not trophic enough, in elderly patients, to obliterate the dead spaces following debridement. Vastus lateralis flap may be contraindicated when the muscle is hypovascular or denervated or in case of a purulent wound [20]. For Suda et al. [3] the main limitation of muscular flaps is the lack of a scoring system to measure the functional deficit after this procedure. Gusenoff et al. proposed an algorithm for the management of complex hip wounds after total hip arthroplasty which demonstrated that muscle flap may be used when the infection involves bone and joint or for the management of delayed wound clousure [16]. This algorithm showed 100% salvage of prosthetic components when early orthoplastic surgery consultation was obtained.

Arnold et al. and Ross et al. [15, 19] describe how all their patients treated with muscular flaps were healed and able to bear weight at final follow-up. Moreover Shieh et al. [8] report a case of infected hip prosthesis treated with pedicled vastus lateralis muscle flap and secondary total hip arthroplasty, with complete infection heling after 2 years follow-up; the patient was also able to walke without any assistant device. Patients treated with vastus lateralis muscle flap by Suda et al. [3] had no evidence of infection at follow-up, they could walke with crutches and had a significant reduction of the pain score (VAS). Within the 24 patients reported by Choa et al. [24], there were 2 flap failures and one flap partial necrosis. At final follow-up 6 patients were still on antibiotics, 21 patients (87.5%) were able to walk but only 5 (20.8%), who had retained prostheses, without aid. All the 4 patients who underwent a gluteus maximus advancement flap for chronic periprosthetic infection, described by Ricciardi [26], ambulated with assistive devices and 3 of them received long-term maintenance antibiotic therapy. Overall, in our review we noted highly positive consensus regarding the effectiveness and safety of muscle flap reconstruction in complex hip prosthetic infection, with 97.9 to 100% healing rate at final follow-up.

Finally, our results must be interpretated considering some limitations. First of all, the number of currently available studies to include and the study samples investigated are relatively small (15 and 220 respectively) in order to draw definitive conclusions and indications. Furthermore, it is necessary to underline the retrospective design of the involved studies and the absence of randomized controlled trials. Anyway, these weak points were potentially offset by our methodology of research and analysis. In fact, this systematic review was conducted in accordance with the PRISMA guidelines as to ensure a comprehensive literature research involving the main electronic databases and based on clear and reliable inclusion and exclusion criteria.

## Conclusions

Given the increasing number of hip joint replacements globally performed every year, a careful evaluation of the optimal management for the treatment of possible complications, remains of great importance. In accordance with the results reported by the different authors in the present review, muscular flaps provide a stable, well vascularized soft-tissue coverage and an antibiotic delivery system for patients with persistent infection after total hip arthroplasty. Further, larger studies, with a randomized controlled design, may consolidate these findings.

## Data Availability

The datasets used and/or analyzed during the current study are available from the corresponding author on reasonable request.

## Abbreviations

THA: Total hip arthroplasty
THR: Total hip replacement
VAS: Visual analog scale
